# Design and Characterization of Piezoresistive Sensors for Non-Planar Surfaces and Pressure Mapping: A Case Study on Kayak Paddle

**DOI:** 10.3390/s24010222

**Published:** 2023-12-30

**Authors:** Abdo-Rahmane Anas Laaraibi, Gurvan Jodin, Corentin Depontailler, Nicolas Bideau, Florence Razan

**Affiliations:** 1 Department of Mechatronics, École Normale Supérieure de Rennes, 35170 Bruz, France; gurvan.jodin@ens-rennes.fr (G.J.); corentin.depontailler@ens-rennes.fr (C.D.); florence.razan@ens-rennes.fr (F.R.); 2 SATIE Laboratory, UMR CNRS 8029, École Normale Supérieure de Rennes, 35170 Bruz, France; 3 OASIS, IETR UMR CNRS 6164, Université de Rennes, 35042 Rennes, France; 4 Movement, Sports and Health (M2S) Laboratory, EA 7470, Université Rennes 2, ENS Rennes, 35170 Bruz, France; nicolas.bideau@univ-rennes2.fr; 5 MIMETIC Team, INRIA Rennes Bretagne Atlantique, 35042 Rennes, France

**Keywords:** non-planar surface, kayak paddle, flexible sensors, piezoresistive sensors, hand pressure, viscoelastic model, Bluetooth Low Energy (BLE), pressure mapping

## Abstract

This article focuses on the design of a sensor system for a non-planar surface, in particular a cylindrical shape, such as a kayak paddle. The main objective is to develop a piezoresistive sensor system to measure the pressure exerted by the hand on the shaft. The study begins with static characterization of the sensors, including dispersion analysis to assess their sensitivity, linearity and measurement range. A calibration process is carried out using a dedicated test bench, and an inverse viscoelastic model is used to establish an accurate relationship between the measured resistance and the corresponding pressure. The sensor system is connected to a data acquisition board equipped with an analog-to-digital converter (ADC) that enables the direct conversion of analog data into digital resistance values. Furthermore, Bluetooth Low Energy (BLE) wireless communication is employed to facilitate data transfer to a computer, enabling a detailed pressure mapping of the kayak paddle and real-time data collection. The calibrated sensors are then tested and validated on the kayak paddle, facilitating the mapping of pressure zones on the paddle surface. This mapping provides information for locating areas of high pressure exertion during kayaker movements.

## 1. Introduction

### 1.1. State of the Art

Flexible and stretchable electronics are a newly developed technology with a broad spectrum of applications. This is due to their components being capable of compression, twisting, and adaptation to complex non-planar surfaces [1]. Currently, wearable electronics have a positive impact on several aspects of daily life, contributing to economic growth and the rapid development of stretchable electronic devices and related manufacturing technologies. Flexible, soft and stretchable electronic devices facilitate the development of next-generation wearable technology, enabling a wide range of applications in healthcare [2], energy harvesting [3], and sports [4].

Flexible pressure sensors employ various sensing mechanisms, including resistivity, piezoelectricity, capacitance, and piezoresistivity, to detect applied forces over an area that will be called pressure in the rest of the paper. Related to this technology, pressure mapping plays a critical role in comprehending human interactions with diverse surfaces, especially in fields such as sports equipment design and biomechanics [5]. The distribution of pressure on non-planar surfaces holds particular significance in activities like kayaking [6], golf [7], and even handlebars for bikes or cars [8], where the shape of the equipment greatly impacts performance [9].

In the world of kayaking, numerous electronic measurement systems come into play to meticulously track athlete’s actions and elevate the prowess of kayakers. A notable example is the work of Klitgaard et al. [10], which delved into the performance of sprint kayak paddling on water. The study specifically focused on the dynamics of leg muscle-generated pushing force, employing a custom-made footrest equipped with two single-point load cells (LCM200 from Futek, Irvine, CA, USA). Their findings underscore the pivotal role played by the rhythmic leg movement in sprint kayak technique, offering valuable insights for coaches and athletes alike in the realm of performance monitoring and enhancement. However, in order to establish a direct link between an athlete’s exertion and boat speed, it becomes imperative to account for the forces applied to the paddle.

Helmer et al. [11] examined the hydrodynamic pressure experienced at a point on the paddle using a force sensor mounted under each blade. Measurements were taken during kayak training sessions to assess paddle technique and efficiency. While the blade pressure sensors could not directly measure the force of the paddle stroke, they provided a synchronized measurement of the paddle’s pull time, enabling characterization of the movement. However, system waterproofing issues were encountered during the trials.

Gomes et al. [12] conducted an investigation into the application of strain gauges directly bonded onto the paddle shaft to analyze time-force curves related to paddle strokes at varying frequencies among elite kayakers. These strain gauges served the purpose of quantifying the forces applied to the paddle. However, it is worth noting that these endeavors carried a significant financial burden, primarily due to the procurement of materials such as ergometers and specialized load cell manufacturing equipment.

Bonaiuto et al. [13] presented the findings of a pilot study aimed at assessing the capabilities of the e-Kayak system, a wireless data acquisition system tailored for performance analysis in flatwater sprint kayaking. The e-Kayak system was employed to measure a range of parameters pertinent to kayaking performance, such as stroke rate, force, and power. This system allows for a comprehensive analysis of kayak propulsion, identifying specific technical flaws in paddling technique. Nevertheless, it is important to acknowledge certain limitations of the study. Notably, it did not delve into the long-term reliability of the e-Kayak system. Furthermore, the authors highlight that, while the e-Kayak system is meticulously designed for kayaking applications, its suitability for other water sports or activities remains a subject of consideration.

Nates et al. [14] introduced a novel six-component paddle force sensor system tailored for measuring the dynamic interaction between a kayaker’s hands and the paddle shaft. This sensor was developed for utilization by elite kayakers during ergometer sessions or on-water paddling. Initial findings underscore the potential of this innovative instrumentation in furnishing invaluable insights to enhance our comprehension of kayaking propulsion. However, it is worth noting that the current process is not ergonomic due to the total mass of 430 g for each sensor, which can cause issues during use. This is despite the design of a specific paddle handle for testing the system. Furthermore, the calibration procedure for the load sensor is complex and needs to be repeated if the sensor is repositioned or if there are variations in bolt tightening.

Other systems have been implemented; Niu et al. [6] introduced an innovative approach to assess kayak paddling performance using a custom-designed paddle equipped with cutting-edge optical fiber technology, specifically fiber Bragg grating strain sensors [15]. This system was engineered for real-time measurement of handle load and blade load distribution during kayak paddling. Results obtained from these fiber optic sensors can reveal that how one grips the handle can be a determining factor in kayaking performance, with notable differences observed between competitive and recreational paddlers. Consequently, accurately assessing the pressure exerted by the hand on the paddle handle poses a significant challenge. However, it is worth noting that despite its conclusiveness, fiber optic technology incurs a substantial cost and includes a lower bandwidth, which can restrict the amount of data transmitted, and an increased sensitivity to temperature changes that can alter the refractive index and degrade performance.

Five commercial alternatives to this theme are listed in the Table 1.

Several commercialized systems primarily focus on measuring the applied force (shaft deformation), cadence, and paddle orientation (Table 1). However, none of these systems specifically address the issue of the load exerted on the handle, particularly the characteristics of the loads on the left and right handles.

### 1.2. Objective of the Present Study

In this context, it becomes imperative to determine this pressure to gain deeper insights into the interaction mechanisms of various biomechanical parameters associated with motor actions, particularly during the execution of critical competitive exercises.

The main objectives of this research are to explore the design and characterization of piezoresistive sensors on non-planar surfaces and to examine how gripping affects boat propulsion efficiency. This paper presents a case study focused on the design and characterization of these piezoresistive sensors, using a kayak paddle as an illustrative example of non-planar surfaces.

The objective of this study is to develop a sensor system featuring a 4 × 4 matrix structure, employing a piezoresistive layer, namely Velostat, between copper electrodes for concurrent measurements at 16 pressure points. Once integrated into the paddle, this system accurately gauges hand pressure on the handle, enabling thorough performance analysis and improvement for kayakers through detailed pressure mapping. The resulting data offers valuable insights into pressure distribution on the paddle surface, fostering a deeper understanding of the kayaker-paddle interaction during various movements.

To achieve this goal, the study comprises several parts. Firstly, the static characterization of the piezoresistive sensors is conducted, encompassing sensitivity, linearity, and measurement range analysis. By evaluating these characteristics, the suitability of the sensors for pressure mapping on non-planar surfaces can be determined. A calibration process is then performed using a dedicated test bench, establishing an accurate relationship between the measured resistance and the corresponding pressure.

These sensors are connected to a data acquisition board equipped with an analog-to-digital converter (ADC) that enables the direct conversion of analog data into digital resistance values. Furthermore, Bluetooth Low Energy (BLE) wireless communication is employed to facilitate data transfer to a computer, enabling a detailed pressure mapping of the kayak paddle and real-time data collection.

In this study, it should be noted that our focus lies on the sensor’s development rather than the utilization of sports results.

## 2. System Description

The primary aim of this section is to design a piezoresistive sensor system capable of accurately measuring the pressure applied to the kayak paddle shaft.

**Figure 1:**
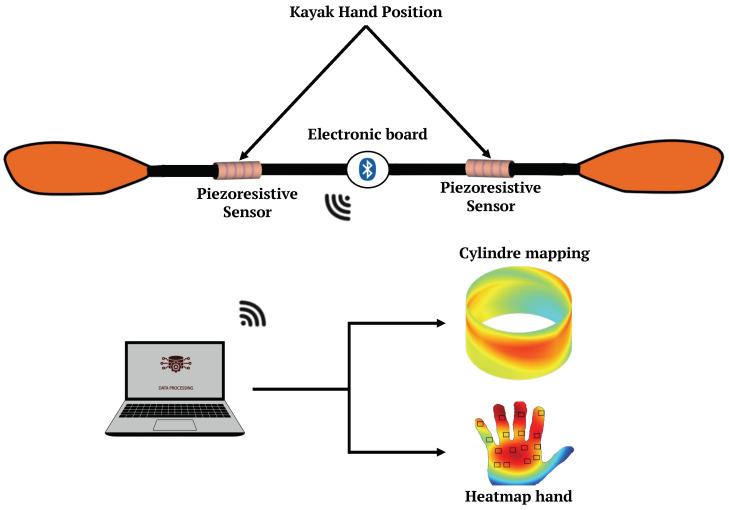
Paddle system with piezoresistive sensors using BLE transmission technology, offering pressure mapping for both the hand and the cylindrical surface.

The overall view of the system is represented by the diagram in Figure 1. This figure illustrates the paddle system, featuring two integrated piezoresistive sensors on the paddle and an electronic board powered by a battery. These sensors are strategically positioned to effectively cover the entire surface where the hand comes into contact with the paddle shaft [21]. Furthermore, the two sensors are independent, as reported in previous studies [12,22], where differences between the two hands were identified. The electronic board includes a microcontroller, BLE radio, and regulator. The data collected by the sensors is transmitted using BLE technology to a computer, ensuring system portability and mobility. This data is then utilized to generate detailed mappings of both the paddle and the kayaker’s hand.

For this study, we have selected the Arduino Nano 33 BLE (Arduino, Ivrea, Italy) due to its advanced features and capabilities for prototyping. It provides the necessary computational power and efficiency for real-time data processing, as well as low energy consumption and BLE connectivity, which are crucial for our wireless data transmission needs. Its compact form factor and lightweight design make it the perfect choice for seamless integration into our sensor system, ensuring minimal disruption to the kayaking experience. Additionally, it comes equipped with an inertial measurement unit (IMU) that includes an accelerometer, gyroscope, and magnetometer, adding substantial value by enhancing precision and paddle orientation during kayaking maneuvers.

## 3. Description of the Piezoresistive Sensor

### 3.1. Working Principle

Building upon our previous research [23], piezoresistive sensors are applied across diverse domains, including medical applications [24], object recognition [25], facial recognition (smile and breath detection [26], and eye blink tracking [27]), and motion monitoring [28]. These sensors are able to measure changes in electrical resistance in response to pressure or strain, a phenomenon arising from the intricate reorganization of charged particles within the material. We focus on two critical physical phenomena: quantum tunneling and percolation. Quantum tunneling assumes a major role when applied pressure modulates the inter-particle distances within the polymer matrix, facilitating electrical interactions among the conducting particles enclosed within Velostat. In contrast, percolation is closely tied to the transition between insulating and conducting states within the material, a transformation induced by varying levels of applied pressure.

The prototype sensor described in this study is constructed on a monolayer of polyolefin impregnated with carbon black, specifically Velostat (3M Electronics division, Saint Paul, MI, USA). It is characterized by high resistivity, measuring less than 500 Ω-cm.

### 3.2. Design of the Piezoresistive Sensor

In this section, we present the sensor that was tested, which is based on a matrix structure, as depicted in reference [25]. The piezoresistive layer is situated between two copper electrodes. To facilitate the measurement of electrical resistance, the copper electrodes are connected to electrical wires.

To ensure optimal contact between the electrodes and the Velostat material, a layer of polyimide, namely Kapton^®^, has been used. This Kapton layer not only improves mechanical strength and electrical insulation, but also minimizes the risk of voltage interference, guaranteeing accurate and precise sensor readings.

**Figure 2:**
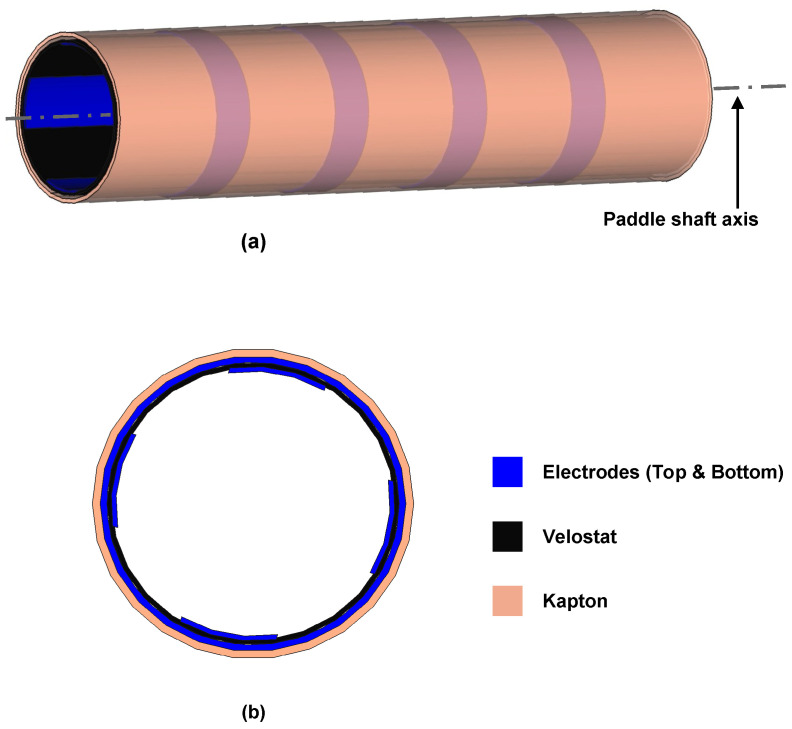
Piezoresistive sensor design–non-planar surface (cylinder) (**a**) and top view of the sensor (**b**).

The sensor under examination boasts a 4 × 4 matrix structure, allowing for concurrent measurements at 16 distinct pressure points, as visually illustrated in Figure 2.

### 3.3. Resistance Measurement Technique

To determine the resistance of these matrix points, we employ a method that involves measuring the resistance between a row and a column, primarily located at their intersection.

**Figure 3:**
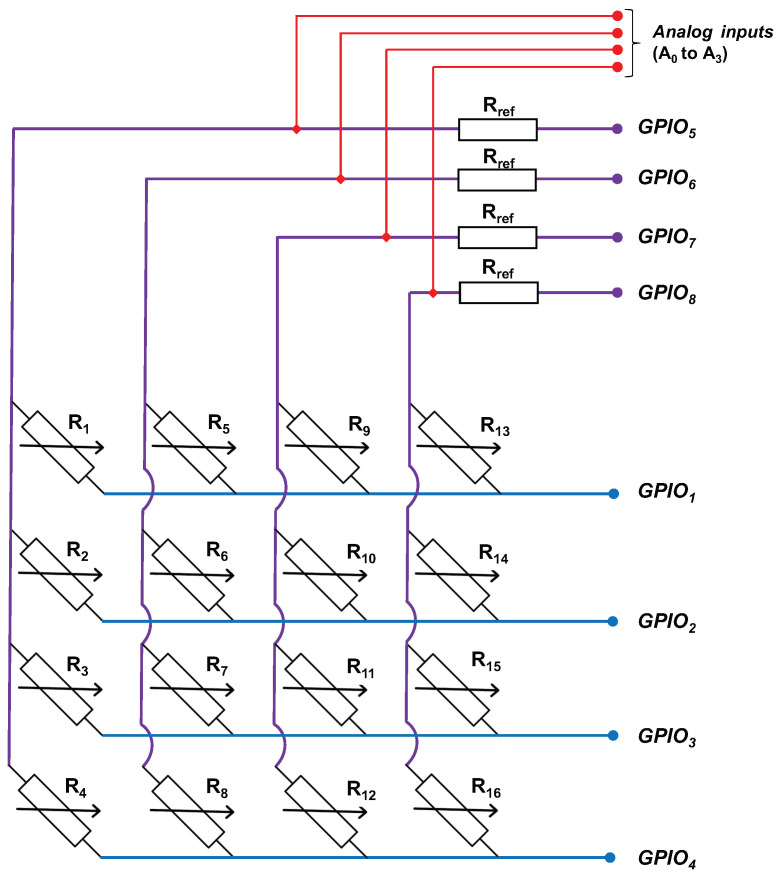
Measuring resistance using GPIO through the voltage divider bridge technique, where $A_{0}$ to $A_{3}$ serve as analog inputs.

This measurement process is illustrated in the electrical diagram shown in Figure 3, where resistance measurements are conducted via General Purpose Input/Output (*GPIO*) ports using a voltage divider bridge. This bridge configuration places the sensor resistance, denoted as $R_{i}$, in series with a fixed, known reference resistor, labeled as $R_{ref}$.

The analog input voltage of *ADC*, denoted as $V_{i}$, is directly linked to the resistance measurements through Equation (1). The pivotal role of the *ADC* lies in its ability to transform the analog voltage into a digital value, making it suitable for subsequent analytical procedures.

(1) $$V_{i}=V_{in}\cdot \frac{R_{i}}{R_{i}+R_{ref}},$$

where, $V_{i}$ represents the voltage at the *i*-th sensor point generated by GPIO [5, 8], $V_{in}$ stands for the supply voltage, $R_{i}$ corresponds to the resistance at the *i*-th sensor point, and $R_{ref}$ denotes the reference resistor. *i* varies within the range [1, 16].

### 3.4. Energy Consumption Analysis of the Sensor

Accurately assessing the energy consumption of the sensor is a fundamental aspect of our study, presented as follows:

- Determining data interrogation time (*t*${}_{\mathrm{on}}$): our initial task involves calculating the data interrogation time *t*${}_{\mathrm{on}}$ for each GPIO port when operating with a sampling interval of 100 ms. This step provides us with insights into the timing aspects of the sensor’s data acquisition process.
- Summation of sensor point power: next, we delve into the specifics of the sensor’s power consumption. We calculate the power consumed at each individual sensor point. This step aims to capture the variations in power draw across different regions of the sensor.
- Average power consumption (*P*${}_{\mathrm{av}}$): to derive an overall picture of the sensor’s energy usage, we sum the power consumed at each sensor point, as determined in the previous step. This summation allows us to obtain *P*${}_{\mathrm{av}}$, as outlined in Equation (2).

(2) $$P_{av}\left(t\right)=\frac{1}{T}\cdot \frac{t_{on}}{N}\cdot \sum_{i=1}^{N}\frac{V_{in}^{2}\left(t\right)}{R_{ref}+R_{i}\left(t\right)},$$

in the context of this equation, *T* signifies the time interval over which the computation of average power is conducted, effectively serving as the temporal scope for the analysis. Meanwhile, *N* denotes the total count of sensors. The parameters such as $V_{in}$, $R_{ref}$, $R_{i}$, and $t_{on}$ have been previously defined in Section 3.3 and discussed in the text above.

## 4. Experimental Setup and Sensor Characterization

### 4.1. Test Bench Configuration

**Figure 4:**
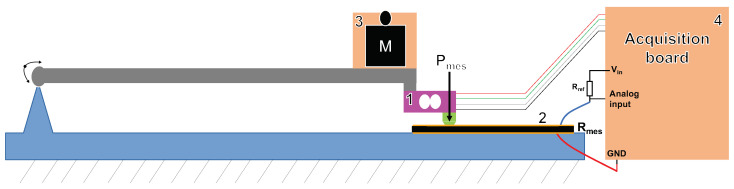
Diagram illustrating the test bench for measuring piezoresistive sensor resistance and pressure force: 1. load cell, 2. piezoresistive sensor, 3. receptacle, and 4. acquisition board.

In this section, we provide a comprehensive description of our designed device for simultaneous measurement of sensor resistance and applied pressure force. Our characterization test bench features a precisely dimensioned 1 × 1 cm${}^{2}$ support target. Crucially, the assembly is guided with precision using a joint mechanism that provides a substantial lever arm, minimizing friction and ensuring smooth vertical translation, as illustrated in Figure 4.

**Figure 5:**
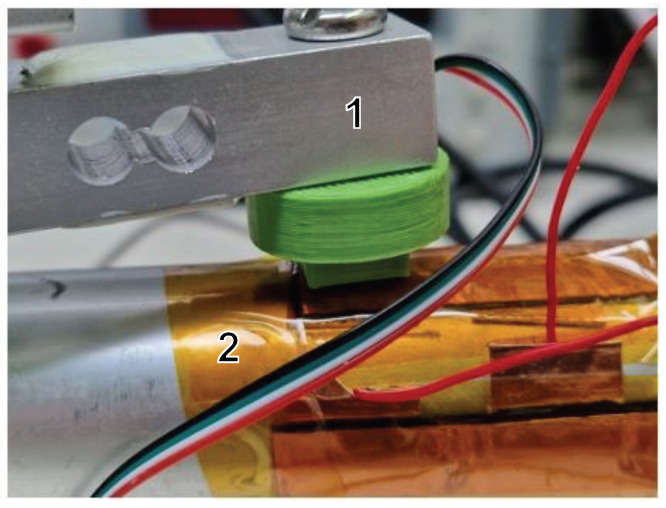
Visualization of the experimental setup for the characterization of the paddle shaft sensor (the number references correspond to those in Figure 4).

This device comprises the following elements: a 500 Newton load cell for measuring the applied pressure (*P*${}_{mes}$), a 3D-printed component with a contact surface of 10 mm${}^{2}$ (PLA) to enable precise and consistent force application to the sensor, and a piezoresistive sensor that was directly integrated into the paddle shaft, as shown in Figure 5. Additionally, it includes a receptacle for weights and an Arduino UNO acquisition card for synchronized data acquisition of resistance (*R*${}_{mes}$). To measure sensor resistance, we implemented a voltage divider, as explained in Section 3.3, using Equation (1).

This designed test setup offers the precision and synchronization required for a detailed characterization and analysis of the sensor response to varying pressure forces.

### 4.2. Characterization Static Methodology

In order to obtain a thorough understanding of the behavior and repeatability of the flexible piezoresistive sensor (80 mm × 90 mm) wrapped around the paddle (Ø29 mm), a static characterization study was conducted. This process utilized a dedicated test bench, as depicted in Figure 5.

The experimental protocol involved progressively applying force to the sensor using weights. After each force application, an 8-minute pause was introduced to allow the sensor to stabilize. During this time, we monitored changes in the sensor’s resistance. Importantly, we repeated this procedure five times on the same sensor, ensuring that each iteration was consistent and reliable.

### 4.3. Viscoelastic Modeling and Dynamic Characterization

**Figure 6:**
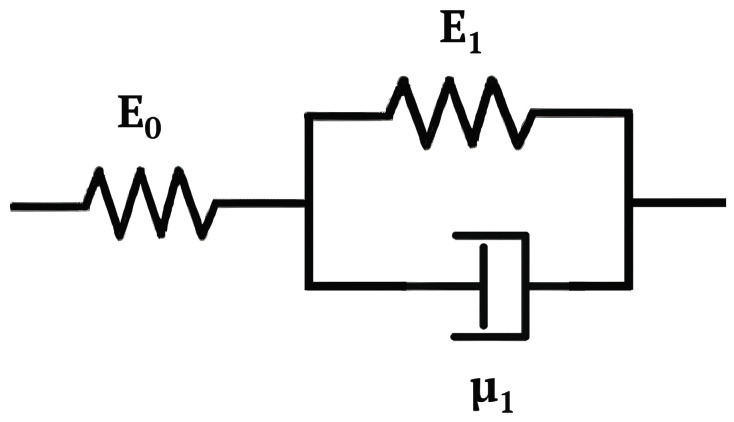
Scheme of the standard linear solid model.

In this section, we delve into viscoelastic modeling and dynamic characterization of the sensor, employing our dedicated test bench. Our primary aim here was to establish a reverse relationship between the sensor’s resistance and the applied pressure, allowing us to estimate applied pressures based on the sensor’s resistance. This modeling employed a standard linear solid (SLS) model, as depicted in Figure 6, and was previously detailed in our earlier work [23].

The algorithm employed in this context, initially introduced by [23], unfolded in two distinct phases. Firstly, it began with the optimization of model parameters (E${}_{0}$, E${}_{1}$, and $\mu_{1}$) to accurately estimate resistance (Direct Model). Subsequently, the parameters optimized in the initial phase were utilized to estimate the applied pressure (Inverse Model). The precise definition of the sensor’s surface area played a pivotal role in this optimization, consistently set at 1 cm${}^{2}$ for each point within the matrix for our study. The algorithm was implemented using MathWorks MATLAB R2023a.

For the dynamic characterization stage, dynamic forces were applied to the sensor using the test bench to assemble our initial dataset, serving as the training dataset. This dataset included the vector of the applied pressure measured by the load cell, the sensor’s resistance, and the vector of time used for optimizing model parameters.

**Figure 7:**
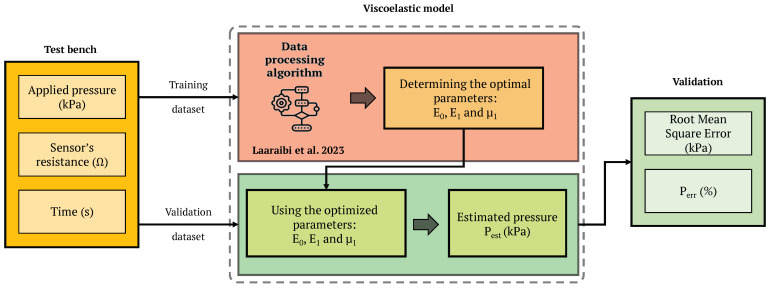
An overview of the viscoelastic modeling process [23].

Once the optimal parameters of the SLS model were determined, we collected multiple validation datasets. These datasets were then subjected to testing within the Inverse Model to validate its parameters and yield precise estimations of the applied pressures. This process was executed for all 16 sensor points, providing a comprehensive assessment of the system’s capabilities, as depicted in Figure 7.

To gauge the accuracy of our estimation model compared to real values, we calculated the Root Mean Square Error (RMSE) between the model’s predictions and the actual values across the entire dataset, as was defined in Equation (3). Additionally, we calculated the percentage error (*P*${}_{err}$) in pressure estimation, following the formula in Equation (4).

(3) $$RMSE=\sqrt{\frac{\sum_{1}^{\tau }{\left(P_{mes}-P_{est}\right)}^{2}}{\tau }}.$$

(4) $$P_{err}\left(\%\right)=100\cdot \frac{P_{est}-P_{mes}}{240}.$$

In these equations, *P*${}_{mes}$ represented the pressure measured by the load cell, *P*${}_{est}$ denoted the pressure estimated by the Inverse Model, and τ signified the measurement period.

The force was standardized to 24 N (i.e., a pressure of 240 kPa over 1 cm${}^{2}$ area) for each sensor, resulting in a cumulative capacity of 384 N for our 16 sensors. This choice was in line with the typical maximum force amplitude reported by Tornberg et al. [22], who observed higher maximum forces (375 N for men in the 1000 m event and 290 N for women in the 500 m event) during on-water measurements conducted with members of the Australian national kayak team.

Once we accurately estimated the applied pressure on the sensor, we could proceed to implement a comprehensive pressure mapping of each hand region, a feature presented in Section 5, enhancing our understanding of sensor performance under varying force levels.

## 5. Results

This section presents the comprehensive characterization of flexible piezoresistive sensors, covering static and dynamic conditions. It details the pressure mapping of hand-induced forces on the paddle shaft and examines the system’s power consumption. Additionally, it explores conductance as a crucial parameter to understand sensor response to pressure changes, highlighting its sensitivity and role in precise force measurements and sensor versatility.

### 5.1. Quasi-Static Characterization

During the initial phase of our study, we conducted a quasi-static characterization of the flexible piezoresistive sensor. This involved applying various weights on the test bench and continuously monitoring the resistance of the sensor in response to the applied pressure.

**Figure 8:**
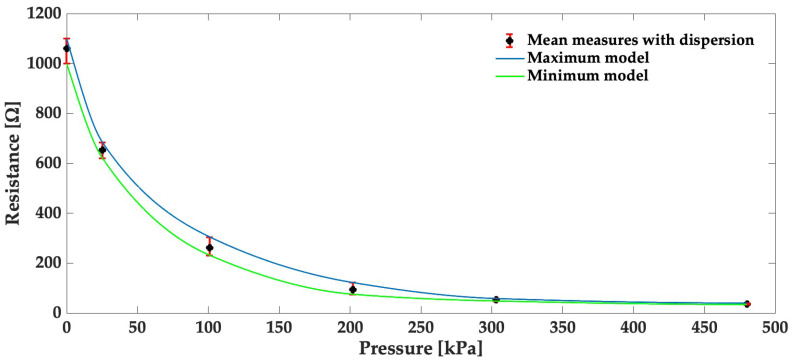
Characteristics of resistance vs. pressure, with error bars indicating minimum and maximum values.

The results of these tests, presented in Figure 8, were obtained from five repeated trials, providing insights into the repeatability and reliability of the sensor’s response. The central black point in Figure 8 signifies the median resistance value, with the lower and upper error bars covering the entire range of measurement values, from the minimum to the maximum.

As the applied pressure increased, the sensor’s resistance decreased, aligning with the behavior of the Velostat material known for its resistance reduction under mechanical pressure. This observation not only underscores the sensor’s consistent response but also demonstrates its repeatability, exhibiting a relative error of 7%.

**Figure 9:**
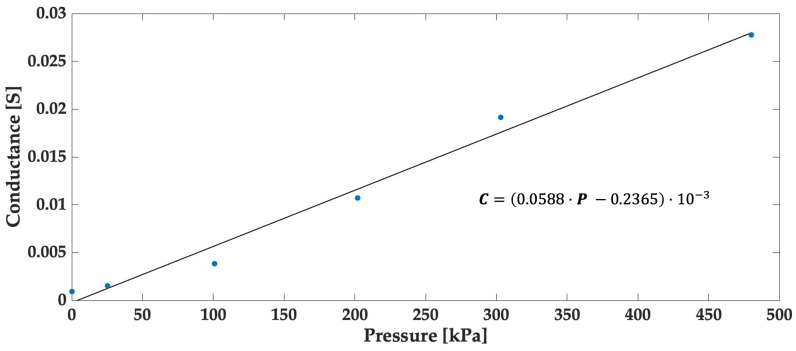
Conductance characteristics vs. pressure, highlighting the best linear fit (black line) with a coefficient of determination ($R^{2}$) = 0.98 for the linear regression model.

Furthermore, we conducted a study on the conductance $G=1/R_{mes}$. Conductance plays a pivotal role in quantifying how the electrical conductivity of the piezoresistive sensor changes in response to pressure variations, as clearly illustrated in Figure 9. Moreover, the analysis of these data enabled the determination of our sensor’s sensitivity, estimated at approximately 58.8 mS/Pa, highlighting the sensor’s excellent responsiveness to pressure changes.

Complementing our quasi-static study, additional investigations involving the same sensor type were conducted to assess hysteresis effect [23].

Following the static characterizations of the piezoresistive sensor, dynamic tests were conducted on the matrix sensor. These dynamic tests revealed a viscoelastic behavior and a memory effect, characteristics not observed during the static characterizations, which are further discussed in Section 5.2.

### 5.2. Dynamic Characterization and Data Processing of the Viscoelastic Model

**Figure 10:**
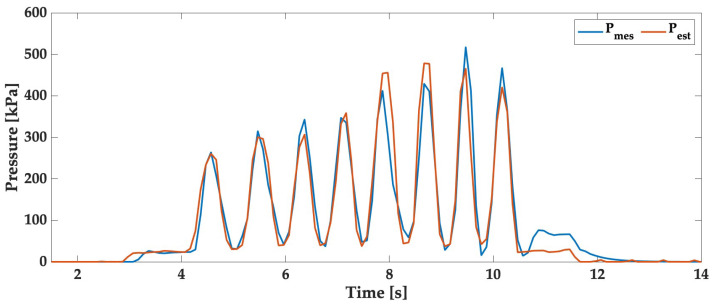
Dynamic pressure estimation using the optimized SLS model: comparison of measured reference and estimated pressure from piezoresistive sensor over time.

In this section, we utilized the algorithm described in Section 4.3 to optimize the parameters of the SLS model using the training dataset sampled consistently at 10 Hz, as shown in Figure 10. This figure illustrates dynamic pressure estimation using the optimized SLS model. In this plot, the blue line represents measured pressure values over time, while the red line represents pressure values estimated by the SLS model. The precision of the model in estimating pressure changes is evident, with an RMSE of 27 kPa for this dataset, using Equation (3). We also observe that our model exhibits good dynamics and closely tracks the actual measurements. However, there is a slight initial delay in the estimation at the beginning and a minor underestimation towards the end.

Using the training data presented in Figure 10, 90% of the errors remain below the 10% threshold, as calculated by Equation (4).

**Figure 11:**
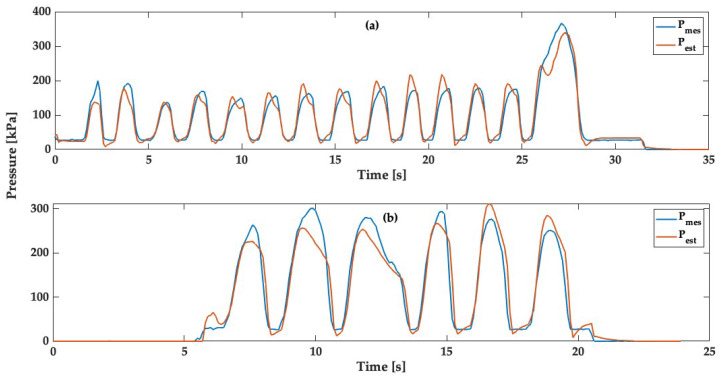
Dynamic pressure estimation for the validation dataset over time; dataset 1 (**a**) and dataset 2 (**b**).

Applying the same methodology, we employed the optimized parameters obtained from the training dataset. Subsequently, these parameters were applied to dataset 1 and dataset 2, as illustrated in Figure 11a and Figure 11b, respectively.

**Figure 12:**
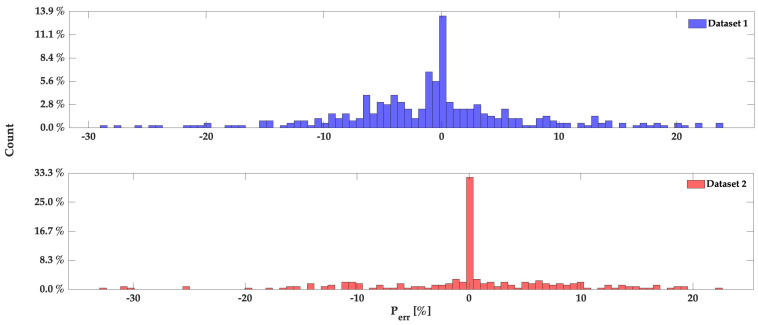
Percentage error of the validation dataset of Figure 11.

Based on the results presented in Figure 11a,b, which are validation data, our inverse algorithm effectively estimates the applied pressure and closely tracks the actual values. However, the algorithm struggles to accurately estimate certain very high peaks, but this is not a significant issue since such cases are infrequent for this kayak scenario (Total Forces < 375 N). The RMSE for both datasets is 25 kPa for dataset 1 and 29 kPa for dataset 2. Additionally, 90% of the errors for both validation datasets are below 15%, as shown in Figure 12. This observation strongly corroborates the accuracy of the proposed model in pressure estimation.

**Figure 13:**
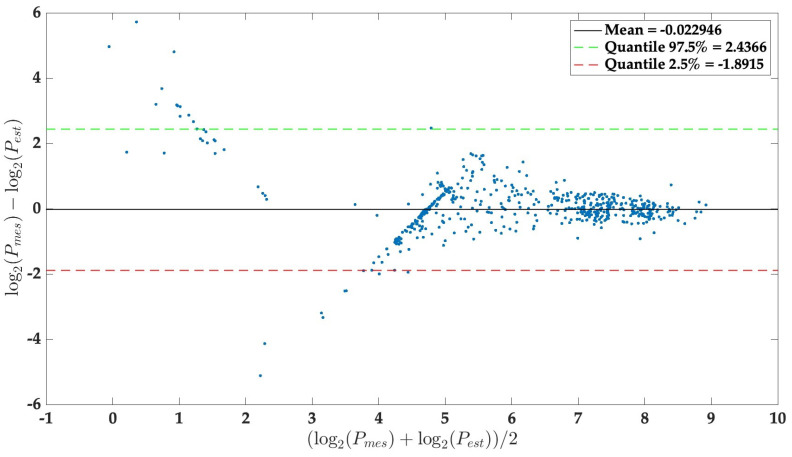
Bland-Altman diagram of the training and validation dataset, depicting the comparison between estimated and reference pressure.

Figure 13 illustrates the difference between pressure estimation (*P*${}_{est}$) and reference pressure measurements (*P*${}_{mes}$) with a Bland–Altman plot. These differences are represented as a function of the amplitude of the measurement (i.e., the average between *P*${}_{mes}$ and *P*${}_{est}$). These amplitudes and deviations are plotted on a logarithmic scale to prevent the larger values from outweighing the smaller ones. The plot includes quantiles representing 95% of the differences between *P*${}_{est}$ and *P*${}_{mes}$.

### 5.3. Paddle and Hand Mapping

With the capability to estimate applied force at each sensor point, we can now generate a comprehensive pressure map of the paddle handle and a corresponding heat map of the hand. This mapping enables us to precisely identify the hand regions undergoing the highest stress during paddle grip. To accomplish this, we employed the ‘v4’ interpolation technique, which utilizes Biharmonic spline interpolation (MATLAB 4 griddata method). Unlike alternative methods, this interpolation does not rely on data triangulation.

**Figure 14:**
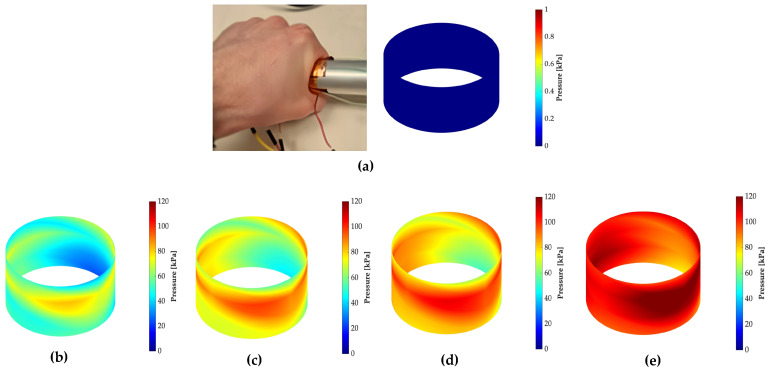
Pressure distribution on the paddle during gripping: (**a**) no applied pressure; (**b**) applied pressure below 60 kPa; (**c**) applied pressure below 90 kPa; (**d**) applied pressure below 100 kPa; (**e**) applied pressure above 100 kPa.

**Figure 15:**
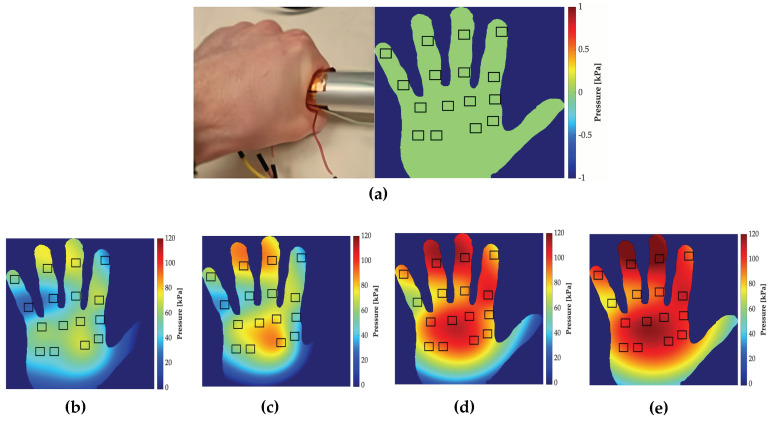
Pressure mapping of the hand: (**a**) no applied pressure; (**b**) applied pressure below 60 kPa; (**c**) applied pressure below 90 kPa; (**d**) applied pressure below 100 kPa; (**e**) applied pressure above 100 kPa.

Figure 14 illustrates the distribution of pressure applied to the paddle handle. It is evident that pressure is primarily concentrated along the entire length of the cylinder, as shown in the green region. This signifies the space between the fingertips and the palm of the hand, which experiences notably lower pressure, as visually represented in Figure 15.

Figure 15 provides a visual representation of this hand mapping at different stages of paddle handle gripping, incorporating data from all 16 sensor points. To position the black squares on the hand, we utilized a planar 4 × 4 sensor and placed it on the hand to determine the location of each point.

At the outset, when the paddle handle is untouched, no pressure registers on the 16 sensor points distributed across the hand’s contact area (Figure 15a). As the paddle handle is gripped, the pressure on the hand gradually increases (Figure 15b–e). Consequently, specific areas of the hand bear more substantial stress compared to others, offering crucial insights into the distribution of pressure during paddle handle gripping. It is important to acknowledge that while this simulation offers valuable qualitative information, a more comprehensive study is needed to precisely analyze the pressure distribution across different hand areas.

These two pressure mappings provide an essential foundation for understanding hand dynamics during paddle handling and offer opportunities for improving kayaker training, as well as minimizing stress concentration on specific areas of both the hand and the paddle handle.

### 5.4. Energy Efficiency Analysis

As explained in Section 3.4, the GPIOs alternate driving the voltage dividers to interrogate all the resistance of the sensor, with an interrogation duration of $t_{on}$ = 12 ms occurring every 100 ms. To save energy, the GPIOs are not driven most of the time. The data presented in Figure 14 indicates a $P_{av}$ = 201.8 μW, this result is a key metric for understanding the sensor’s energy efficiency, as given by Equation (2). Notably, the sensor exhibits low average power consumption, accounting for a mere 0.36% of the total system power usage. This underscores the sensor’s ideal suitability for applications where conserving power resources is of paramount importance.

## 6. Discussion

This paper presented a case study that focused on the design and characterization of piezoresistive sensors tailored for non-planar surfaces, using a kayak paddle as an illustrative example. The sensor’s 4 × 4 matrix structure allowed for the simultaneous measurement of pressure at 16 different points. This sensor exhibited a sensitivity of 58.8 mS/Pa for a range of 0 to 500 kPa. This increased sensitivity meant that slight variations in pressure resulted in more significant changes in the electrical resistance of this sensor. The measurements of resistance were conducted via GPIO ports using a voltage divider bridge.

The integration of these sensors into the paddle enabled comprehensive pressure mapping, providing valuable insights into kayaker’s performance. A significant advantage of the system is the low average power consumption of the sensor, which is 208.1 μW at 3.3 V using the Arduino Nano 33 BLE. This choice due to its advanced features, crucial for real-time data processing, a vital element in kayaking performance analysis. Furthermore, its BLE connectivity supported wireless data transmission, enhancing mobility during paddle training and performance analysis.

Afterward, the design and setup of our characterization test bench are based on a lever arm and a load cell connected to a 3D-printed component, further enhancing the precision of force application on a 1 × 1 cm${}^{2}$ area. This test bench allowed us to measure the force applied to the sensor, consequently generating the electrical resistance measurement data. In the same context, we conducted a quasi-static characterization of the sensor applying various weights on the test bench while continuously monitoring the sensor’s resistance in response to the applied pressure. The results, illustrated in Figure 8, were collected through five repeated trials, yielding valuable insights into the sensor’s ability for repeatability and reliability. This not only highlights the sensor’s consistent behavior but also emphasizes its remarkable repeatability, with a relative error of approximately 7%.

Moving on to viscoelastic modeling and dynamic characterization using the SLS model, we established a relationship between sensor resistance and applied pressure. This allowed us to estimate applied pressures based on resistance. The algorithm employed in this context, initially introduced by [23], proceeded in two distinct phases. First, it optimized model parameters (E_0_, E_1_, and $\mu_{1}$) to ensure accurate resistance estimation (direct model). Subsequently, the parameters optimized in the initial phase were used to estimate applied pressure (inverse model). The algorithm was implemented using MathWorks MATLAB R2023a.

Subsequently, we applied this algorithm to optimize the parameters of the SLS model using the initial training dataset sampled at 10 Hz. Figure 10 illustrates dynamic pressure estimation using the optimized SLS model, yielding an RMSE of approximately 27 kPa. We applied the same methodology to implement the optimized parameters from the initial dataset to other test datasets, as shown in Figure 11. The results depicted in these figures confirm the effectiveness of our inverse algorithm in estimating applied pressure, with 90% of the errors for both validation datasets staying below 15%. These findings strongly support the accuracy of the proposed model for pressure estimation, with an RMSE of around 29 kPa.

However, during the dynamic tests, the model exhibited a slight initial delay in estimating pressure at the test’s onset and a minor underestimation towards the end. Additionally, the algorithm faced challenges in accurately estimating exceptionally high peaks. Fortunately, this is not a significant concern, given the infrequent occurrence of such cases in the kayak scenario where total forces remain below 375 N [22]. Despite these inaccuracies, this is a step forward for sports applications. In fact, the proposed system is capable of accurately extracting pressure waveforms, and extracting the frequency and amplitude of movement is possible like existing sensors, with more precise information on pressure distribution and dynamics.

The Bland–Altman plot analysis is a method used to assess the bias between mean differences and to estimate an agreement interval, encompassing 95% of the differences between the estimated pressure (*P*${}_{est}$) and the reference pressure (*P*${}_{mes}$). By applying a logarithmic scale, we ensured that larger values did not dominate the analysis over smaller ones. This graph highlights the disparities and sheds light on the piezoresistive sensor’s performance in relation to the amplitude of the applied pressure. In this representation (Figure 13), we can observe that the tested pressures remain relatively consistent across the pressure range. However, it is noteworthy that the graph indicates a tendency for the estimated pressure to underestimate the actual values, particularly at lower pressure levels.

After pressure estimation, we successfully generated a comprehensive pressure map of the paddle handle and a corresponding heat map of the hand. This mapping enabled us to illustrate the distribution of pressure applied to the paddle handle and accurately identify the hand regions experiencing the highest stress during paddle grip. To accomplish this, we applied the ’v4’ interpolation technique, which utilizes Biharmonic spline interpolation. Nonetheless, it is crucial to acknowledge the limitations of these pressure mappings. While they offer valuable insights, the data they provide is aesthetic and qualitative rather than quantitative in nature.

## 7. Conclusions

In conclusion, this study of flexible piezoresistive sensors designed for non-planar surfaces has provided valuable insights into their behavior and adaptability, particularly in the context of kayak paddle. Quasi-static characterizations demonstrated repeatability, with a relative error of approximately 7%. Additionally, in dynamic conditions, the sensor exhibited reliability and repeatability. The use of a viscoelastic model established a relationship between sensor resistance and applied pressure, resulting in an average RMSE of around 27 kPa for the sensor, including the transducer, acquisition system, and processing algorithm. Furthermore, this study confirms the sensor’s recovery characteristic, allowing it to quickly and effectively return to its initial state after exposure to external stimuli. Despite some minor limitations involving initial delays and underestimation of high pressures, these sensors exhibit great potential in various applications for non-planar surfaces. Moreover, the energy efficiency analysis confirmed the low power consumption of the sensors, thus validating our initial hypothesis.

Looking ahead, we are considering further enhancements to the microcontroller to achieve even greater energy efficiency. Additionally, our future research agenda includes conducting in situ tests (on-water conditions) to assess sensor performance under such conditions, with plans to incorporate athlete trials for comprehensive evaluation.

## Figures and Tables

**Table 1:** An overview of various commercialized kayaking systems.

Systems	Operating Principles	Wireless Data Transmission
	Handle Force	
Paddlemate [16]	Distance	Bluetooth
	Speed	
Vaaka Cadence [17]	Paddle Stroke Frequency	Bluetooth and ANT+
Kayak Power Meter [18]	Paddle force	Bluetooth
Motionize [19]	Distance	Bluetooth
	Paddle Stroke Frequency	
Allegro Kayak [20]	Kayak Cadence	ANT+
	Stroke Rate	

## Data Availability

The data presented in this study are available on request from the corresponding author.

## Abbreviations

The following abbreviations are used in this manuscript:

ADC	Analog-to-Digital Converter
BLE	Bluetooth Low Energy
IMU	inertial measurement unit
GPS	Global Positioning System
GPIO	General Purpose Input/Output
SLS	Standard Linear Solid
RMSE	Root Mean Square Error
